# Push-out bond strength evaluation of endodontic bioceramic sealer (Bioroot RCS) after final irrigation with different intracanal irrigants: A comparative in vitro study

**DOI:** 10.6026/973206300221611

**Published:** 2026-03-31

**Authors:** Varsha Sam, V.P Prabhath Singh, Anju Varughese

**Affiliations:** 1 Department of Conservative Dentistry and Endodontics, Amrita School of Dentistry, Amrita Vishwa Vidyapeetham, Kochi, Kerala, India

**Keywords:** Bioceramic sealer, Bio Root RCS, citric acid, ethylenediamine tetra acetic acid, octenidine dihydrochloride

## Abstract

Successful root canal therapy also depends on an apical seal that further inhibits microbial re-infection in addition to biomechanical debridement. Therefore, it is of interest to evaluate bioceramic root canal sealer (Bioroot RCS) push-out bond strength difference using various intracanal final irrigants. The apical portion of 75 extracted mandibular premolars were enlarged till a size of 30.4% and segregated into five groups based on the final irrigant as Group 1 - 10% Citric acid, Group 2 -0.2% Chitosan, Group 3 -0.1% Octenidine dihydrochloride, Group 4 -17% Ethylenediamine tetra acetic acid and Group 5 - distilled water (control group). 3mm sample sections were obtained from the coronal third of the specimens following obturation using Bioroot RCS sealer and gutta-percha and further push out bond strength was evaluated. The various mode of failure (Adhesive, Cohesive and Mixed) were observed under a stereomicroscope of 20X magnification following the test. Thus, we show that 10% citric acid when used as the final irrigant can enhance the bond of the bioceramic sealer (Bioroot RCS) with the radicular dentin which may lead to a favourable endodontic outcome.

## Background:

Successful root canal therapy is achieved not only by biomechanical debridement but also depends on apical seal which further prevents microbial re-infection. Endodontic sealers adaption to dentinal walls has its influence on apical seal. Root canal sealers vary in composition, including zinc oxide-eugenol (ZOE), epoxy resin (AH Plus), methacrylate resin, calcium hydroxide (Seal apex), glass ionomer, and bioceramic-based materials, each with distinct properties and limitations, such as solubility, cytotoxicity, dimensional instability, and moisture sensitivity. ZOE based sealers are used as traditional sealers due to their high antimicrobial activity by eugenol release but have its potential limitations due to its cytotoxicity, porosity and shrinkage [1, 2]. Epoxy resins are advised for enhanced sealing; however, they pose challenges due to their cytotoxic properties, which arise from the emission of monomers during the polymerization process [3]. Sealapex is commonly utilized in retreatment cases because of its alkaline pH that facilitates periapical healing, despite its limitations in dimensional stability. Glass ionomer-based sealers (*e.g.*, Ketac-Endo) offer chemical adhesion to dentin and fluoride release but are technique-sensitive and relatively brittle, which may affect long-term performance [1, 4]. High sealing ability, dimensional stability and biocompatibility are the main properties to be considered for flawless seal. One such widely used material is BioRoot RCS, a calcium silicate-based material. It has distinct advantage over conventional resin-based sealers due to chemical bonding with dentin by forming hydroxyapatite chains which gives long term adhesion and sealing [2]. The smear layer, which is formed during the biomechanical preparation of the root canal, influences the adhesion of sealers and obstructs penetration because of the blockage of dentinal tubules, thereby affecting bond strength and sealing efficiency [5]. Usage of Root canal irrigants clears smear layers and increases better adaptability of sealers. Despite its propensity for dentin erosion and cytotoxicity, 17% ethylenediaminetetraacetic acid (EDTA) is used in standard clinical practice because of its chelating properties. Therefore, alternative agents like 10% citric acid, with its acidic nature that removes smear layer while preserving dentin microstructure and 0.2% chitosan, a biopolymer derivative, with its chelating, antimicrobial property and biocompatible properties are used [6, 7]. 0.1% Octenidine dihydrochloride, a bispyridine antiseptic is another widely used endodontic irrigant due to antimicrobial properties though its influence on sealer adhesion remains uncertain [8]. Distilled water, commonly used as a control in studies, lacks both smear layer removal and antibacterial effects and establish baseline comparisons. Bond strength of sealers needs to be assessed for their clinical adaptability. Amongst all, push-out bond strength test, measures the exertion force to dislodge a sealer from dentin by providing simulation to induce functional stress [9]. Resin-based sealers adhere by covalent bond formation with collagen fibrils in dentin matrix whereas Bioceramic sealers, primarily through micromechanical interlocking and infiltration zones within dentin [10]. Both differ in their bonding mechanism. Most studies shows adaptability of resin based sealers but sparse literature is evident related to bioceramic sealers and its adhesion strength. Therefore, it is of interest to compare bioceramic sealer (Bioroot RCS) push out bond strength using various intracanal final irrigants.

## Methodology:

This study was approved by the Institutional Ethics Committee (ECASM-AIMS-2021-194) at the Amrita Institute of Medical Sciences and Research Centre. The sample preparation was done in the department of Conservative Dentistry and Endodontics: Amrita School of Dentistry. A total of 75 freshly extracted human mandibular first premolars were collected based on inclusion criteria of with single-root canal anatomy and fully formed apices. Teeth with anatomical variations, root caries, cracks, fractures, resorption defects and previous endodontic treatment were excluded. Radiographic analysis was done to confirm canal anatomy. As illustrated in (Figure 1a - see PDF), teeth were decoronated using a diamond disk to preserve a standard root length of 14 mm [11]. Working length was established using size 10K, 15K and 20K stainless steel files (Mani, Japan). Apical preparation was performed using HyFlex CM files in the sequence 20/.04, 25/.04, 20/.06, and 30/.04, used consecutively to the working length. During preparation, recapitulation of canals using 20K-file was done followed by irrigation with 5.25% sodium hypochlorite (NaOCl) delivered via a 30G side-vented irrigation needle [9]. Random sampling done and categorised into five groups (n = 15). Group 1 received 10% citric acid (Swakit Biotech, Bangalore) [12] Group 2 received 0.2% chitosan (Swakit Biotech, Bangalore) [9] Group 3 received 0.1% octenidine dihydrochloride (Octenisept, Germany) [8] Group 4 received 17% EDTA (Waldent) [13] and Group 5, serving as the control group, received distilled water (Cero) All the samples were irrigated with respective irrigants of 5ml quantity. Following this, the canals were obturated using BioRoot RCS (Septodont, USA) with gutta-percha (30.04 taper) via the lateral condensation technique. Samples were kept for seven days at 37°C in phosphate-buffered saline post coronal seal procedure [9].

## Push-out testing:

Post incubation, each root was sectioned horizontally from the coronal third with diamond disk under water cooling to achieve a thickness of 3 mm, as seen in (Figure 1b - see PDF) [14]. A customized jig which is depicted in (Figures 2a & b - see PDF) was fabricated to mount the acrylic blocks containing the specimens for push-out bond strength testing. A universal testing machine (Tinius Olsen, Germany) as in (Figure 2c - see PDF) was used to apply force via a cylindrical plunger measuring 0.5 mm diameter in apico-coronal direction by maintaining a crosshead speed of 1.3 mm/min [14]. The cylindrical plunger with a diameter of 0.5 mm was aligned centrally on the filling material within the root canal to ensure adhesion with the obturation material, avoiding the surrounding dentin. Bond failure was indicated by the dislodgement of the bioceramic sealer and a corresponding abrupt drop on the load-deflection curve and results were analysed using Horizon.

After the procedure following were measured such as, Maximum debonding load measured in Newtons (N). Bond strength in Megapascals using the formula σ = F / SL, Where, F is the load in Newtons, SL = π (R + r) h, with R and r as the coronal and apical radii respectively and h the slice thickness [15]. Failure modes were assessed as shown in (Figure 2d - see PDF) under a stereomicroscope at 20x magnification and classified as adhesive, cohesive, mixed [16].

## Statistical analysis:

Statistical analysis was performed using IBM SPSS version 20.00, Chicago, USA. Normal Data distribution was assessed by Kolmogorov Smirnov test. ANOVA was used to evaluate statistical significance of bioceramic sealer (BioRoot RCS) bond strength to dentinal walls among five experimental groups, followed by Boneferroni Post Hoc test to identify intergroup differences. Statistical significance considered with p<0.05.

## Results:

Citric acid had the highest push-out bond strength (3.4467±0.3042), while distilled water, the control group, had the lowest value (1.724±0.74903) (Table 1 - see PDF). The Octenidine Dihydrochloride group had the lowest mean (2.2747±0.57572) out of the four test groups. When compared to distilled water, ANOVA showed a statistically significant difference (p≤0.05) between EDTA, chitosan, and citric acid (Table 2 - see PDF). Citric acid significantly increased the push-out bond strength of BioRoot RCS as compared to all other groups, including chitosan (p = 0.025), Octenidine dihydrochloride (p < 0.001), EDTA (p < 0.001), and pure water (p < 0.001), according to the Bonferroni multiple comparison test. However, there was no statistically significant difference when the Octenidine dihydrochloride group was compared with the control group (Table 3 - see PDF). The maximum percentage of adhesive failures were contributed by the EDTA group (80%), cohesive failures by the Citric acid group (60%) and Mixed failures by the Octenidine dihydrochloride (33%) and Citric acid group (33%) respectively. It could be concluded the majority of the failure mode seen in the test groups were adhesive in nature. The citric acid group was an exception with cohesive failure (9/15) seen predominantly. All the test groups showed the mixed failure as the second most common type of failure (Figure 3 - see PDF).

## Discussion:

The declining use of resin-based endodontic sealers as a consequence of increased cytotoxicity has taken bioceramic sealers to the forefront. With their superior biocompatibility and bioactive properties, bioceramic sealers promise to fulfil the majority of the criteria of an ideal root canal sealer [17, 18]. Bioroot RCS root canal sealer is a tricalcium-silicate endodontic sealer modified with povidone and polycarboxylate used in the present study. The sealer is known for its calcium release, apatite-forming ability, and strong alkalinizing activity. The prolonged mineralizing ion release of the calcium silicate-based endodontic sealer may improve the sealing ability of the obturation materials compared to the other sealers [19]. The organic and inorganic debris produced following root canal instrumentation, collectively known as the smear layer covers the dentin and blocks the dentinal tubule orifice. This can obstruct the penetration of the sealers and compromise its satisfactory seal to the root dentin leading to microleakage [20, 21]. Complementing root canal irrigation with endodontic sealers can amplify the benefits of sealers by the removal of the smear layer. Sodium hypochlorite is the routinely used root canal irrigant in endodontic therapy. It is a powerful organic solvent with proteolytic and broad-spectrum antibacterial activity. However, supplemental final irrigation with a chelating agent such as EDTA needs to be employed to treat the inorganic portion of the smear layer [22, 23- 24]. 10% Citric acid shows better biocompatibility compared to 17% EDTA, on top of antimicrobial activity and smear removal [25]. In current study, 10% citric acid showed highest mean values of push out strength. This runs counter to a study by Carvahlo *et al.* that found no statistically significant difference in the push-out bond strength of calcium-silicate-based endodontic sealers when employing final irrigant groups that contained 17% EDTA, 2.25% Pera acetic acid, and 10% Citric acid [26]. Chitosan, a biopolymer with chelating and antimicrobial properties, also showed favourable results. Unlike EDTA, Chitosan does not degrade collagen fibrils and has the ability to abolish smear layer with minimal erosive effect on the dentin [27]. 0.2% Chitosan solution was used over 0.37% as the final irrigant in the present study. This was because a much greater erosive effect was seen on the radicular dentin using 0.37% Chitosan solution with the same cleaning efficacy as that of 0.2% Chitosan solution [28]. There are various studies in the literature comparing the push-out bond strength of a bioceramic sealer using 0.2% Chitosan and 17% EDTA as root canal irrigants in favour of the results obtained in our study [29, 30]. Inspite of being a potent antimicrobial agent, 0.1% Octenidine dihydrochloride exhibited the lowest bond strength owing to lack of chelation property and incompetency to remove smear layer that further hinders sealer penetration and bonding. The control group (distilled water) also had lower bond strength, due to failure in smear layer removal. In addition to the above properties, higher incidence of cohesive failures showed in the citric acid group, indicating better internal strength of the sealer interface. Weaker sealer-dentin bonding is inferred in Octenidine and control groups due to high frequency of adhesive failure in these groups. A recent update on the use of citric acid in endodontic treatment showed the irrigant to be effective in smear layer removal from the coronal and middle root thirds improving its effect when combined with manual dynamic activation. However, there was no agreement regarding the effect of citric acid on sealer adhesion and adaptation to root canal walls in the systematic review due to heterogeneity within studies [31]. Nevertheless, *in-vitro* design and the absence of long-standing mechanical or thermal aging confine the present study. Further clinical research is to be carried out to validate these results. To summarise, final irrigants play a crucial role in the action of bioceramic sealers with dentin adhesion. Better root canal optimization occurs by considering irrigants like citric acid and chitosan and comparative studies have to be carried among new bioceramic materials like EndoSequence BC Sealer, and BioRoot RCS over conventional AH Plus Sealer for efficacy evaluation [32].

## Conclusion:

We show the importance of the final irrigant in determining the adhesion of BioRoot RCS to radicular dentin and its influence in increasing the push out bond strength of the sealer. Citric acid demonstrated better results and Octenidine showed poor bond strength. Better irrigants increases micro mechanical retention of bio sealers. So, clinicians should incorporate irrigants with better chelating properties such as citric acid or chitosan for successful endodontic treatment. Furtherance, research is recommended with larger sample sizes with inclusion of multi rooted tooth as well.
